# Animal Breed Composition Is Associated With the Hindgut Microbiota Structure and β-Lactam Resistance in the Multibreed Angus-Brahman Herd

**DOI:** 10.3389/fmicb.2019.01846

**Published:** 2019-08-13

**Authors:** Peixin Fan, Corwin D. Nelson, J. Danny Driver, Mauricio A. Elzo, Kwangcheol Casey Jeong

**Affiliations:** ^1^Emerging Pathogens Institute, University of Florida, Gainesville, FL, United States; ^2^Department of Animal Sciences, Institute of Food and Agricultural Sciences, University of Florida, Gainesville, FL, United States

**Keywords:** breed composition, gut microbiota, intramuscular fat, antimicrobial resistance, β-lactam resistance

## Abstract

Antibiotics have been widely used in livestock to treat and prevent bacterial diseases. However, use of antibiotics has led to the emergence of antibiotic resistant microorganisms (ARMs) in food animals. Due to the decreased efficacy of antibiotics, alternatives to antibiotics that can reduce infectious diseases in food animals to enhance animal health and growth performance are urgently required. Here, we show that animal genetics is associated with the hindgut microbiome, which is related to fat deposition and beta-lactam resistance in the gastrointestinal tract. We investigated the hindgut microbiota structure in 95 postweaning heifers belonging to the unique multibreed Angus-Brahman herd with breed composition ranging from 100% Angus to 100% Brahman. The hindgut microbial composition of postweaning heifers differed among breed groups. The mucin-degrading bacterium *Akkermansia* known for promoting energy expenditure was enriched in Brahman calves that contained less intramuscular fat content, while butyrate-producing bacterium *Faecalibacterium* was linearly positively correlated with Angus proportion. Moreover, the higher relative abundance of beta-lactam resistant genes including ampC gene and arcA gene was associated with the greater Brahman proportion. As the first study aimed at understanding changes in hindgut microbiota among beef cattle with linear gradient of breed composition and its association with marbling in meat, our results suggest that the effects of animal genetics on the gut microbiota structure is associated with fat deposition and potentially a factor affecting the gut antimicrobial resistance.

## Introduction

Antibiotic resistance has become a huge threat to public health as well as one of the major causes of global human mortality over the past few years (Ventola, 2015; Ferri et al., 2017). The failure to successfully treat infectious diseases caused by antibiotic resistant microorganisms (ARMs) has resulted in at least 23,000 deaths in the United States annually estimated by the Center for Disease Control (CDC, 2013). It is well accepted that the emergence and rapid dissemination of antibiotic resistance are primarily due to excessive and inappropriate use of antibiotics, which serve as a selective pressure for the emergence of ARMs (Holmes et al., 2016; Bengtsson-Palme et al., 2018). Antibiotics used in agricultural settings account for 50–80% of the antibiotics produced in the United States to prevent or treat animal diseases (Bartlett et al., 2013; Cully, 2014). The main risks associated with the extensive use of antibiotics in agricultural settings is the spread of antimicrobial resistance among food-producing animals, then transmission into humans through food chains (Thanner et al., 2016). Therefore, it is urgent to develop alternatives to antibiotics mitigating ARMs in livestock to enhance animal health and growth performance.

Although antibiotics contribute greatly to antimicrobial resistance, the natural environment is also a reservoir of antibiotic resistome, an aspect that has received little attention. In previous studies, we isolated cefotaxime (a third-generation cephalosporin) resistant bacteria (CRB) from cattle raised without antibiotic use (Mir et al., 2018), supporting the idea that natural cefotaxime resistance may persist in the gastrointestinal tract of animals. Moreover, associations between the abundance of specific gut commensal bacteria and the prevalence of CRB were detected (Mir et al., 2018), suggesting a potential impact of gastrointestinal ecosystem on the antimicrobial resistance. Different bacterial species differ in their susceptibility to antibiotics. For example, bacteria that inhabit environments with highly toxic compounds, produced by plants, often have genomes that include large numbers of biodegradative enzymes and efflux pump encoding genes (Martinez, 2008; Allen et al., 2010). It has been suggested that the gut microbiota harbors a diverse reservoir of antibiotic resistance genes (Sommer et al., 2010), and the antimicrobial resistance genes become more diverse along with the development of gut microbiota during the early stage of life in humans and animals (Penders et al., 2013; Lu et al., 2014). However, the relationship between variation in gut microbiota driven by different gastrointestinal environments and antimicrobial resistance profiles has been largely remained unknown.

In the southern region of the United States, most beef cattle contain a certain percentage of Brahman (*Bos indicus* origin) to cope with hot and humid environmental conditions (Elzo et al., 2012). However, meat from Brahman cattle is less desirable because of low marbling content, smaller ribeye area, and less tenderness compared to the meat from Angus (Johnson et al., 1990; Elzo et al., 2012, 2013), suggesting a substantial discrepancy in nutrient metabolism between the two breeds. In addition, Brahman cattle have the exceptional ability to utilize low quality feed due to their unique digestive system (Hunter and Siebert, 1985). For example, *B. indicus* cattle can transfer more urea to the digestive tract per day, and harbor a greater rumen protozoal population involved in rumen microbial lysis than *Bos taurus* cattle (Hegarty, 2004). Therefore, the substrates provided to the hindgut microbiota of Angus and Brahman are likely distinct.

Although the rumen harvest 85 to 90% of the gross energy available, fermentation in the large intestine supplies 10 to 15% of energy to adult cattle (Delaval, 2002), suggesting the role of hindgut bacteria on animal growth and energy harvest. ARMs in feces of livestock can be directly released to the environment, thereby having a higher risk of transmission to human compared to those colonized in the foregut. Our previous study reported that Brahman cattle harbored less *E. coli* O157 in the hindgut compared to Angus cattle (Jeon et al., 2013), suggesting the effects of animal breed on bacterial colonization. Based on these observations, we propose the hypothesis that animal genetics influences the hindgut microbiota structure, which is associated with antimicrobial resistance and animal growth. The study was designed to investigate the relationship of animal genetics with hindgut microbiota and antimicrobial resistance, as well as the association between hindgut microbiota, animal weight gain, and intramuscular fat. Here, we used a unique multibreed Angus-Brahman (MAB) herd with breed composition ranging gradually from 100% Angus to 100% Brahman, as an animal model.

## Materials and Methods

### Animal Genetic Background

The postweaning heifers in this study belonged to the MAB herd at the University of Florida. The MAB herd was established in 1988 for long-term genetic studies in beef cattle (Elzo and Wakeman, 1998). Calves were assigned to six breed groups (BGs) according to the following breed composition ranges based on pedigree: BG1 (*n* = 15) = 100 to 80% of Angus, 0 to 20% of Brahman; BG2 (*n* = 20) = 79 to 60% of Angus, 21 to 40% of Brahman; BG3 (*n* = 13) = 62.5% of Angus, 37.5% of Brahman, BG4 (*n* = 18) = 59 to 40% of Angus, 41 to 60% of Brahman, BG5 (*n* = 14) = 39 to 20% of Angus, 61 to 80% of Brahman, and BG6 (*n* = 15) = 19 to 0% of Angus, 81 to 100% of Brahman. Mating in the MAB herd followed a diallel design, where sires from six BGs (BG1: *n* = 5, BG2: *n* = 2, BG3: *n* = 4, BG4: *n* = 4, BG5: *n* = 2, BG6: *n* = 5) were mated to dams from same six BGs (BG1: *n* = 21, BG2: *n* = 19, BG3: *n* = 12, BG4: *n* = 15, BG5: *n* = 15, BG6: *n* = 13) (Supplementary Table S1).

### Animal Management and Diet

**FIGURE 1:**
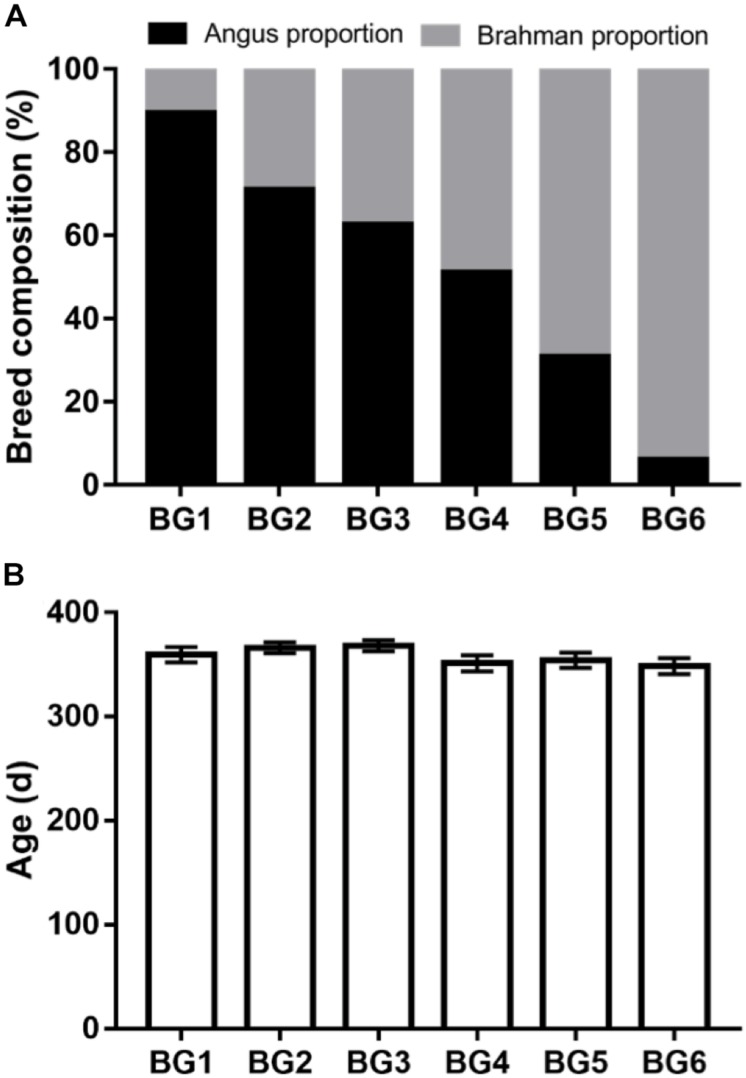
Genetic background and age information of the multibreed Angus-Brahman multibreed (MAB) postweaning heifers. Angus and Brahman proportion of the postweaning heifers among six breed groups (BGs) **(A)**. Heifers were divided into six BGs based on their breed composition. The breed composition of BG1 to BG6 ranged from 100% Angus to 100% Brahman. The age of postweaning heifers among six BGs **(B)**. *n*_BG1_ = 15, *n*_BG2_ = 20, *n*_BG3_ = 13, *n*_BG4_ = 18, *n*_BG5_ = 14, *n*_BG6_ = 15.

The calves in the MAB herd were born and kept at the Beef Research Unit (BRU) at the University of Florida (Gainesville, FL, United States) with no exposure to sub-therapeutic antimicrobials. Ninety-five MAB postweaning heifers (age: 358 ± 28 days) were used in this study. The average of breed composition and age of heifers in each BG are shown in Figure 1. All heifers were kept together in the same pen on a bahiagrass (*Paspalum notatum*) pasture after weaning in August 2016. Additionally, heifers were fed Creep Hazen concentrate (12% crude protein, 2% crude fat, 14% crude fiber; Hillandale quality feeds, LLC, Lake Butler, FL, United States), bahiagrass hay, and had *ad libitum* access to a mineral supplement (UF University Special Custom Winter Mineral, University of Florida, Animal Sciences Department, Gainesville, FL, United States) (Supplementary Table S2).

### Animal Growth Performance and Intramuscular Fat Content

Weight of calves was measured at weaning and 16 weeks after weaning. Percent of intramuscular fat was measured using an Aloka 500 ultrasound system (Hitachi Aloka Medical, Ltd., United States) at the same time as the 16-wk weight was measured. Ultrasound images were analyzed with UICS Scanning Software by Walter and Associates, LLC (Ames, Iowa, United States).

### Fecal Samples Collection and Processing

Fecal samples were collected as previously described with minor modification (Mir et al., 2018). Briefly, sterile cotton swabs were applied to collect fecal samples from the rectal anal junction of 95 postweaning MAB heifers 16 weeks after weaning. Swabs with fecal samples were placed in 15 mL conical tubes on ice and were transported to the laboratory the same day for further processing. Swab samples were resuspended in 2 mL of Luria-Bertani (LB) broth and 2 mL of 30% glycerol, split into four 2 mL tubes and frozen in an ultra-low freezer at −80°C.

### 16S rRNA Gene Sequencing

Genomic DNA was extracted from each 500 μL fecal samples using the QIAamp PowerFecal DNA kit (Qiagen, United States). The concentration and purity of the DNA was measured using a Nanodrop instrument (Thermo Fisher Scientific, United States). The DNA library was prepared and sequenced as described (Kozich et al., 2013). Briefly, the V4 region of the 16S rRNA gene was amplified by polymerase chain reaction (PCR) with dual-index primers (Kozich et al., 2013) and a high fidelity polymerase (AccuPrime, Invitrogen, United States) following conditions: 95°C for 5 min; 30 cycles at 95°C for 30 s, 55°C for 30 s, 72°C for 1 min; and 72°C for 5 min. The amplicons were purified and normalized in equimolar amounts using the SequalPrep plate normalization kit (Invitrogen, United States). Equal amounts of amplicons from each sample were pooled to construct the DNA library. The fragment size and concentration of DNA library were determined by tape station and Kapa qPCR (Kapa Biosystems, United States). Final DNA library (600 μL 6pmol/L library) was loaded into MiSeq v2, 2 × 250 cycle cartridge (Illumina, United States), and were sequenced using the Illumina MiSeq platform in at the Interdisciplinary Center for Biotechnology Research (ICBR) at the University of Florida.

### Microbial Community Analysis

Demultiplexed R1 and R2 sequencing read files were obtained from the Illumina BaseSpace website and analyzed with the Quantitative Insights into Microbial Ecology (QIIME) pipeline (version 1.9.0) (Caporaso et al., 2010). Paired-end reads were assembled with the scripts multiple_join_paired_ends.py and multiple_split_libraries_fastq.py. Chimeric sequences were identified and filtered using the usearch61 algorithm implemented in QIIME with the script identify_chimeric_seqs.py. Sequences were clustered into Operational Taxonomic Units (OTUs) with 99% identity and classified into the taxonomical levels based on Silva 132 database^1^. The sequencing data were normalized to 13,310 (the lowest sequencing depth among samples) with the script single_rarefaction.sh. Alpha diversity (Chao 1 and Shannon index), β-diversity (weighted UniFrac distance), and relative abundance of bacteria at different taxonomical levels (from phylum to genus level) were analyzed on the normalized OTU table with the scripts: alpha_diversity.py, beta_diversity_through_plots.py, and summarize_taxa_ through_plots.py, respectively. Analysis of similarities (ANOSIM) was used to detect statistical differences of UniFrac distance metric with the script compare_categories.py.

### Co-occurrence Network Analysis

To predict bacteria-bacteria interactions in hindgut microbiota, co-occurrence patterns of core bacterial taxa that are present in at least 50% of samples were evaluated in the network interface using pairwise Spearman’s rank correlations based on bacterial relative abundance (Geng et al., 2014). Briefly, the Spearman rank correlation between the relative abundance of each two bacteria was analyzed using Hmisc 3.9-3 package within the R software. The raw *P*-values were corrected using the FDR method in the p.adjust within the R package. Each co-occurring pair had a Spearman rank correlation coefficient above 0.30, with an FDR-corrected significance level under 0.05. The network was visualized using the Fruchterman Reingold algorithms in the interactive platform Gephi^2^. Nodes in the network represent different bacterial taxa and edges indicate significant correlations among nodes. The size of the nodes represents numbers of connection with other bacterial taxa, and the thickness of the edges indicates the strength of the correlation.

### Functional Prediction of the Gut Microbiome

Functional capacity of the gut microbial community was predicted using PICRUSt (phylogenetic investigation of communities by reconstruction of unobserved states) online Galaxy version^3^ (Langille et al., 2013). The closed reference OTU table was generated by picking OTU against the 13 August 2013 Greengenes database. Normalization of copy numbers, metagenome prediction and function categorization based on Kyoto Encyclopedia of Genes and Genomes (KEGG) pathways were conducted according to the standard analysis process.

### Shotgun Metagenomic Sequencing and Downstream Analysis

Extracted DNA aliquots from BG1 and BG6 fecal samples were pooled together within each breed group, respectively. The library for two DNA pools was prepared according to the Nextera XT protocol, and the metagenomic sequencing was performed using the MiSeq Reagent Kit v2 through the Illumina MiSeq platform.

Raw data files from shotgun sequencing were subjected to quality control using Trimmomatic online Galaxy version (Bolger et al., 2014). Sequences with their length shorter than 50 bases and quality score lower than 20 were trimmed for further analysis. The trimmed fastq files were demultiplexed using QIIME and uploaded to the MG-RAST server (Meyer et al., 2008). In MG-RAST, artificial replicate sequences produced by sequencing artifacts and host specific species sequences (*B. taurus*, UMD v3.0) were removed. Annotation was conducted using the KEGG orthology (KO), with a maximum *e*-value of 1 × 10^–5^ and a minimum identity cutoff and alignment length of 60% and 15 bp, respectively. The sequence counts were normalized to 136,878.

### Quantitative PCR

Real-time qPCR was applied to quantify *amp*C, *arc*A, and the 16S rRNA gene in DNA extracted from all the fecal samples. The qPCR assays were performed using the SsoAdvanced Universal SYBR Green Supermix (Bio-Rad, United States) on the CFX96 Touch^TM^ Real-Time PCR Detection System (Bio-Rad, United States). The real-time qPCR program was as follow: initial denaturing at 95°C for 5 min, followed by 40 cycles of 10 s at 95°C, 30 s at different annealing temperatures, and 30 s at 72°C. The fluorescence data were acquired at 72°C, and the final melting curve was constructed with temperature ramping up from 65 to 95°C. Standard curves for were prepared using the genomic DNA of *E. coli* Jeong9592 (Teng et al., 2019). Five-fold serial diluted calibration curve of each gene was tested in triplicate on the same PCR plate. The primer sets and annealing temperatures were as described in Supplementary Table S3.

### Detection of Ampicillin Resistant Bacteria

The culture-based method was used to count the total bacteria and detect the ampicillin resistant bacteria (ARB) in the fecal samples from BG1 and BG6. The resuspension solution of fecal samples were plated on tryptic soy agar at various dilutions in LB broth (10^0^ to 10^–2^) for counting total bacteria and onto tryptic soy agar containing 50 μg/ml ampicillin (BD, United States) for counting ARB (Eriksson et al., 1965). The plates were incubated overnight at 37°C. After 18 h, bacterial colonies were counted.

### Statistical Analysis

The normal distribution of variables was assessed using the Shapiro-Wilk’s test in R software (Rstudio, 2018). When distributions were non-normal, values were log-transformed. For the relative abundance of certain core bacterial taxa that were not present in all the samples, a small numeric constant (half of the detection limit: 0.000038) was added to all values before logarithm transformation.

Multiple linear regressions were applied to analyze the effects of Brahman proportion, age and their interaction on weight gain, intramuscular fat percent, the relative abundance of core bacteria (present in more than 50% of samples) and microbial function using RStudio Version 1.1456 (Rstudio, 2018) as follows:

$$\text{Δ}{\text{Y}}_{\text{i}}\;=\;{\text{β}}_{0}\;+\;{\text{β}}_{1}\;\times \;{\text{X}}_{\text{1i}}\;+\;{\text{β}}_{2}\;\times \;{\text{X}}_{\text{2i}}\;+\;{\text{β}}_{3}\;\times \;({\text{X}}_{\text{1i}}\;\times \;{\text{X}}_{\text{2i}})\;+\;{\text{u}}_{\text{i}},$$

ΔYi: dependent variables (weight gain, intramuscular fat percent, the relative abundance of core bacteria or microbial function, concentration of 16S rRNA gene or antimicrobial resistant genes detected by qPCR); X_1__i_: Age in days; X_2__i_: Brahman proportion, X1i × X2i: interaction of age and Brahman proportion.

To detect whether specific hindgut commensal bacteria contributed to weight gain and intramuscular fat, the relative abundance of core bacteria was added to the multiple linear regression model as another independent variable together with age and Brahman proportion using RStudio Version 1.1456 (Rstudio, 2018) as follows:

$$\text{Δ}{\text{Y}}_{\text{j}}\;=\;{\text{β}}_{0}\;+\;{\text{β}}_{1}\;\times \;{\text{X}}_{\text{1j}}\;+\;{\text{β}}_{2}\;\times \;{\text{X}}_{\text{2j}}\;+\;{\text{β}}_{3}\;\times \;{\text{X}}_{\text{3j}}\;+\;{\text{u}}_{\text{j}},$$

ΔYj: dependent variables (weight gain or intramuscular fat percent); X_1__j_: age in days; X_2__j_: Brahman proportion, X3j: relative abundance of core bacteria.

Differences in Chao 1 and Shannon index among groups were analyzed using the one-way analysis of variance (ANOVA) followed by Tukey’s HSD test for pairwise comparison of multiple means. Correlations between breed composition and prevalence of OTUs (present in more than 50% of sample within at least one BG) were assessed by Pearson correlation coefficients using SAS-JMP. Differences in bacterial colony counts between BG1 and BG6 were analyzed by Student’s *t* test. All these tests were conducted using SAS-JMP (JMP, v12.0.1, SAS Institute, United States). *P* value < 0.05 was considered as statistically significant, and 0.05 < *P* value < 0.1 was considered as tendency.

## Results

### Growth Performance of Postweaning MAB Heifers

**FIGURE 2:**
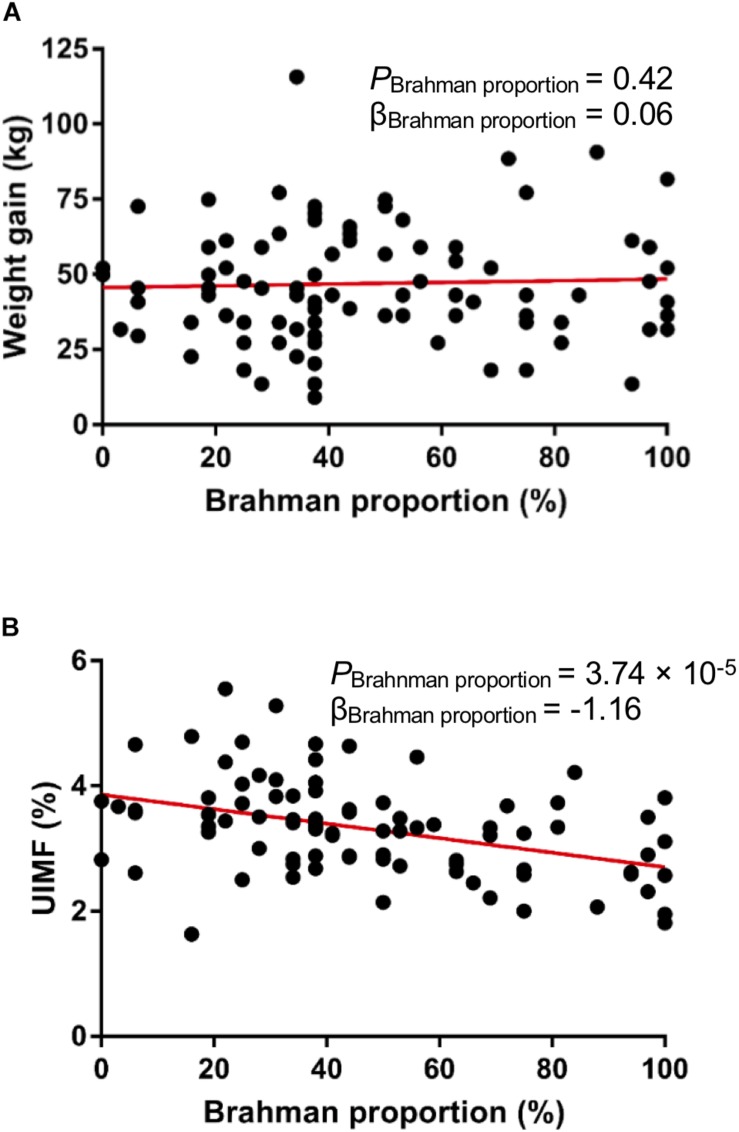
Animal growth performance of the MAB postweaning heifers. There is no significant correlation between breed composition and weight gain **(A)**. Ultrasound percent of intramuscular fat (UIMF) was negatively correlated with Brahman proportion **(B)**.

Multibreed Angus-Brahman calves in this study were weaned on the same day and kept together in one pen after weaning to remove the potentially confounding with factors of interest. We did not observe a significant linear association between breed composition and weight gain after 16-week of weaning (Figure 2A, *P* = 0.42), indicating that the growth rate of the MAB heifers was not influenced by breed composition after weaning. Generally, meat from Angus cattle contains more marbling than meat from Brahman cattle (Elzo et al., 2012). In agreement with this, a significant negative association between Brahman proportion and percent of intramuscular fat measured by the ultrasound system existed in these MAB heifers (Figure 2B, *P* = 3.74 × 10^–5^).

### Overall Hindgut Microbiota Composition of the Postweanng MAB Heifers

**FIGURE 3:**
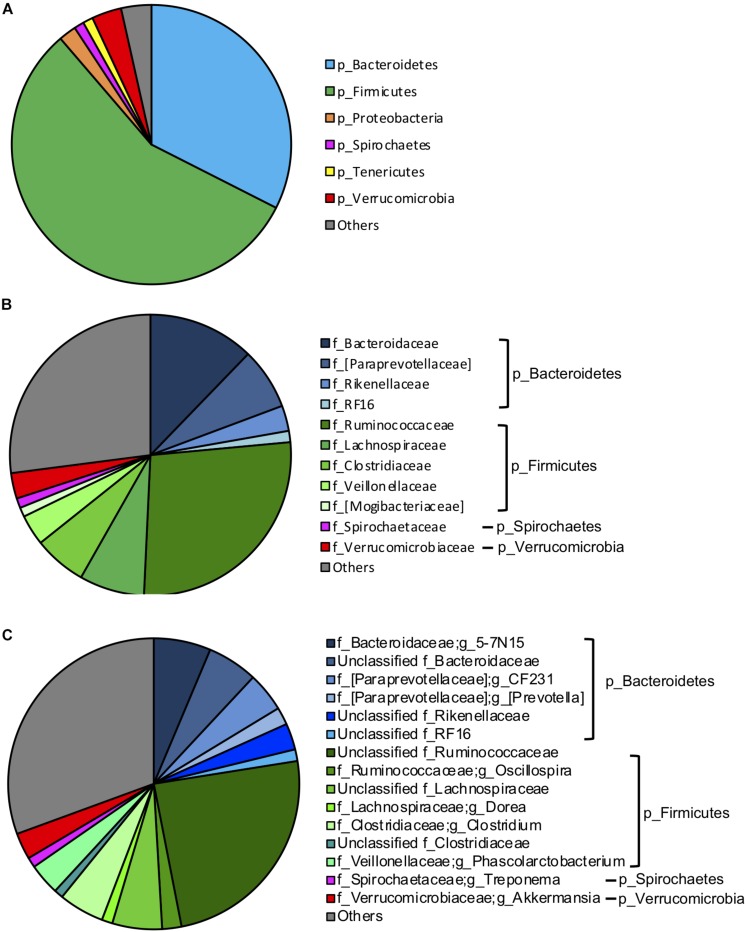
Gut microbiota composition of the MAB postweaning heifers. The dominant (1% of the total bacterial community) phyla **(A)**, families **(B)** and genera **(C)** in the 95 fecal samples of the postweaning heifer.

To investigate the influence of breed composition on the hindgut microbiota, and the contribution of hindgut microbiota to animal growth, we collected fecal samples from the 95 postweaning MAB heifers raised on pasture. Illumina Miseq sequencing of the V4 regions of bacterial 16S rRNA genes was conducted with DNA extracted from these fecal samples. A total of 2,879,231 high-quality reads were yielded. The sequences were normalized to 13,310 reads and grouped into an average of 1,294 OTUs per sample. Firmicutes (56.23%) and Bacteroidetes (32.47%) were the two dominant phyla in the hindgut microbiota of the postweaning MAB heifers (Figure 3A). Other predominant phyla accounted for more than 1% of the hindgut microbial community were Verrucomicrobia (3.44%), Proteobacteria (2.03%), Tenericutes (1.17%) and Spirochaetes (1.15%) (Figure 3A).

At the family and genus levels, Ruminococcaceae (27.21%) in the phylum Firmicutes was the most abundant classified bacterial family (Figure 3B). The majority of Ruminococcaceae was unclassified at the genus level (24.44%), and the only classified genus belonging to this family that occupied more than 1% of the hindgut microbiota was *Oscillospira* (2.14%) (Figure 3C). Bacteroidaceae in the phylum Bacteroidetes was the second most abundant classified bacterial family, consisting mainly *5–7N15* (6.35%) and unclassified genera (5.60%) (Figures 3B,C). Other dominant classified families that accounted for more than 1% in the phylum Bacteroidetes included [Paraprevotellaceae] (7.08%), which primarily contained two genera *CF231* (4.38%) and [*Prevotella*] (1.94%), Rikenellaceae (2.93%) and RF16 (1.29%). Lachnospiraceae (7.48%), mainly including unclassified genera (5.52%) and *Dorea* (1.19%); Clostridiaceae (6.06%), mainly including *Clostridium* (5.05%); Veillonellaceae (3.53%), mainly including *Phascolarctobacterium* (3.50%) and [Mogibacteriaceae] (1.08%) were other abundant families in phylum Firmicutes. Spirochaetaceae (1.12%), including *Treponema* (1.12%) and Verrucomicrobiaceae (2.94%), including *Akkermansia* (2.94%) were dominant families in phyla of Spirochaetes and Verrucomicrobia, respectively (Figures 3B,C).

### Effects of Breed Composition on the Hindgut Microbiota Structure of the Postweaning MAB Heifers

**FIGURE 4:**
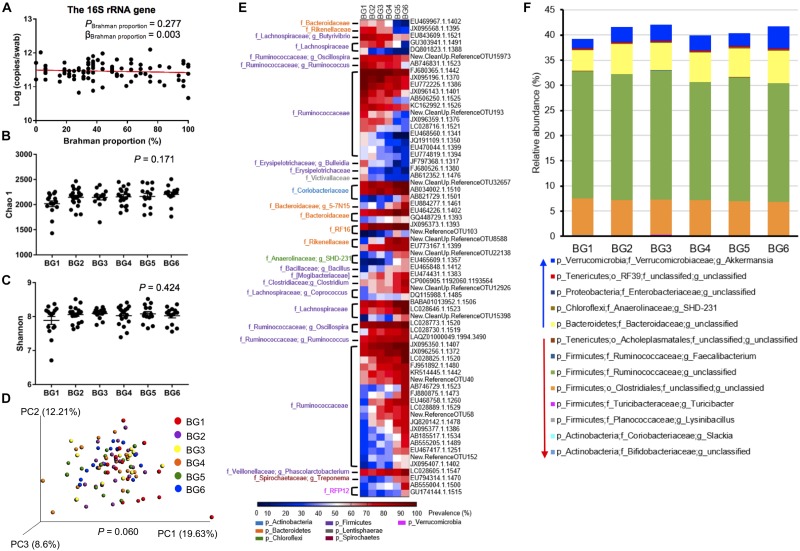
The difference in gut microbiota composition of postweaning heifers among six BGs. The concentration of 16S rRNA gene copy numbers among 95 heifers **(A)**. For alpha diversity, there is no significant difference in both Chao 1 **(B)** and Shannon **(C)** index among six BGs by one-way analysis of variance (ANOVA) followed by Tukey’s HSD test for pairwise comparison of multiple means. However, for β-diversity, the PCoA plot based on weighted uniFrac community distance showing the separation of postweaning heifers among BGs compared by analysis of similarities (ANOSIM) **(D)**. The heatmaps represent OTU prevalence among six BGs. Only OTUs that were at least classified at the family level and with their prevalence showing a significant positive correlation (*P* < 0.01) with breed composition were included in the heatmaps **(E)**. The relative abundances of core (identified in at least 50% of the samples) bacterial taxa that were linearly influenced by breed composition **(F)**.

The 16S rRNA gene copy numbers per swab was detected by qPCR to determine the hindgut bacterial concentration among the 95 heifers. The 16S rRNA gene concentration varied from 4.65 × 10^10^ to 1.25 × 10^12^ per swab, and was not significantly associated with breed composition (Figure 4A, *P* = 0.277). To explore the dynamics of the hindgut microbiota composition in the MAB heifers of different breed composition, the alpha diversity of 6 BGs was evaluated. Both Chao 1 (*P* = 0.1705), reflecting species richness, and Shannon indexes (*P* = 0.4240), reflecting both species richness and evenness, did not show significant differences among BGs (Figures 4B,C). To further detect whether identical and uniform proportions of bacteria colonize the hindgut of postweaning heifers with different breed composition, weighted UniFrac distance, accounting for similarities in both presence and abundance of bacteria was compared using ANOSIM. As shown in the PCoA plot, the microbiota structure tended to be different among BGs (Figure 4D, *P* = 0.060). In the PCoA plot, the separation between samples belonging to BG1 and BG6, having the farthest genetic distance at breed level, was apparent (Supplementary Figure S1), indicating the existence of host genetic impact on hindgut microbiota structure.

To identify bacteria with their presence linearly associated with breed composition, correlation between the prevalence of OTU and Brahman proportion was analyzed. Among the 1272 OTUs that were present in more than 50% of the samples in at least one BG, the prevalence of 101 OTUs showed significant correlation with breed composition (Supplementary Table S4). The prevalence of 68 OTUs classified at least at the family level was strongly linearly correlated with breed composition (R^2^ > 0.7), including 23 OTUs that showed higher prevalence with greater Angus proportion, whereas the other 45 OTUs showed higher prevalence with greater Brahman proportion (Figure 4E). More than half of these OTUs (36 OTUs) belonged to Ruminococcaceae family. Notably, OTUs that were assigned to the identical bacterial family or genus did not always have a consistently opposite or negative correlation with breed composition.

To detect whether the bacterial distribution was linearly influenced by breed composition, we used a multiple linear regression model that included Brahman proportion of heifers, age in days, and interaction between Brahman proportion and age in days, as three independent variables. As a result, among the 85 core bacterial taxa that were present in at least 50% of the samples, the relative abundance of 13 taxa showed significant linear associations with breed composition (Figure 4F, Supplementary Table S5). The breed-associated bacteria occupied 40% of the total hindgut microbial community (Figure 4F). The relative abundances of *Slackia* (*P* = 7.75 × 10^–5^) and unclassified Bifidobacteriaceae (*P* = 0.000411), belonging to *Actinobacteria*, and five Firmicutes bacteria including *Lysinibacillus* (*P* = 0.00177), *Turicibacter* (*P* = *0.0426*), unclassified Clostridiales (*P* = 0.0429), unclassified Ruminococcaceae (*P* = 0.00743), and *Faecalibacterium* (*P* = 0.00296) and one *Tenericutes* bacteria, the unclassified Acholeplasmatales (*P* = 0.0261), were all linearly positively associated with Angus proportion (Figure 4F, Supplementary Table S5). Conversely, unclassified Bacteroidaceae (*P* = 0.00342), SHD-231 (*P* = 0.001072), unclassified Enterobacteriaceae (*P* = 0.0425), unclassified RF39 (*P* = 0.0256) and *Akkermansia* (*P* = 0.00244) were enriched in postweaning heifers with greater Brahman proportion (Figure 4F).

### Associations Between Hindgut Microbiota and Animal Growth

To detect whether the hindgut microbiota composition was associated with variation in weight gain and intramuscular fat content, the relative abundance of core bacteria was added as an additional independent variable to Brahman proportion and age in the multiple linear regression model. As shown in the Table 1, the relative abundance of unclassified Coriobacteriaceae (*P* = 0.00226), *p-75-a5* (*P* = 0.0303) and SHD-231 (*P* = 0.0435) showed positive linear association with weight gain, while the relative abundance of unclassified Dehalobacteriaceae (*P* = 0.00298) and unclassified YS2 (*P* = 0.0178) were negatively linearly associated with weight gain. In addition, the relative abundance of unclassified [Mogibacteriaceae] (*P* = 0.00448) and *Succiniclasticum* (*P* = 0.048) were positively linearly associated with intramuscular fat content.

**TABLE 1:** The association between the log transformed relative abundance of bacteria and animal phenotypes.

**Phenotypes**	**Bacterial taxa**	**β_Bacteria_**	***P* value**
Weight gain	p_Actinobacteria;c_Coriobacteriia;o_Coriobacteriales;f_Coriobacteriaceae;g_unclassified	28.66	0.00226
	p_Firmicutes;c_Erysipelotrichi;o_Erysipelotrichales;f_Erysipelotrichaceae;g_p-75-a5	12.99	0.0303
	p_Chloroflexi;c_Anaerolineae;o_Anaerolineales;f_Anaerolinaceae;g_SHD-231	8.36	0.0435
	p_Firmicutes;c_Clostridia;o_Clostridiales;f_Dehalobacteriaceae;g_unclassified	–13.13	0.00298
	p_Cyanobacteria;c_4C0d-2;o_YS2;f_unclassified;g_unclassified	–8.45	0.0178
Intramuscular fat	p_Firmicutes;c_Clostridia;o_Clostridiales;f_[Mogibacteriaceae];g_unclassified	1.82	0.00448
	p_Firmicutes;c_Clostridia;o_Clostridiales;f_Veillonellaceae;g_Succiniclasticum	0.75	0.0484

### Co-occurrence Network Analysis

**FIGURE 5:**
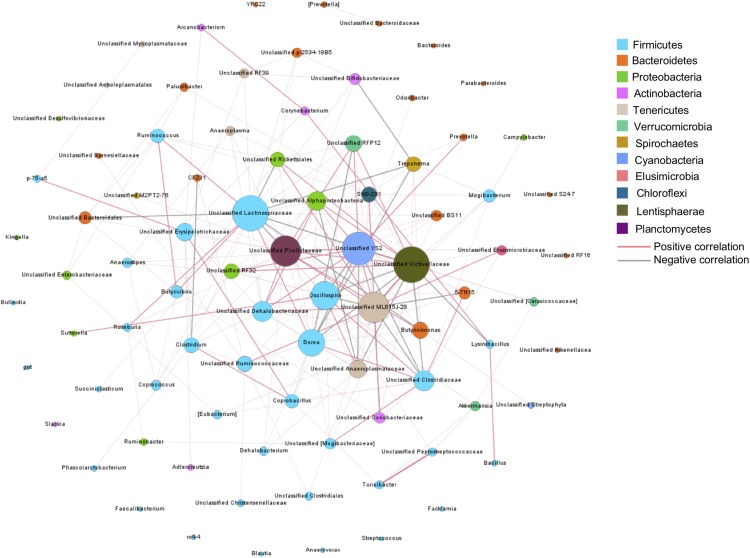
Co-occurrence bacterial network. Each co-occurring pair has an absolute Spearman rank correlation above 0.3 with an FDR-corrected significance level under 0.05. Dot size represents the number of connections with other taxa. Thickness of lines represents the strength of the relatedness.

To understand bacteria-bacteria interactions and identify potential factors affecting these interactions, a co-occurrence network analysis was conducted on the core bacterial taxa, presented in at least 50% of samples. In total, 205 connections with significant Spearman ranking correlations were detected (*P*_adjust_ < 0.05, *r*_s_ > 0.3 or *r*_s_ < −0.3) among 85 core bacterial taxa (Figure 5). Unclassified Victivallaceae and Lachnospiraceae were the hubs of the network, with the largest number of interactions with other core bacteria (*n* = 24). Unclassified Pirellulaceae, YS2, and ML615J-28 also had more than 20 connections with other bacteria. Among these five bacteria, unclassified Victivallaceae and ML615J-28 were influenced by age. Unclassified Lachnospiraceae was affected by age and breed interaction and YS2 was associated with weight gain. Of the 13 breed-associated bacteria, two negative correlations were detected, between *Akkermansia* and *Turicibacter* and between unclassified RF39 and Bifidobacteriacea, suggesting that breed composition may affect the competitive interactions among these bacteria.

### Effects of Breed Composition on the Microbial Metabolic Function Profiles

To detect differences in microbial function, especially metabolic functions influenced by breed composition, the abundance of microbial genes involved in metabolic pathways were predicted using PICRUSt (phylogenetic investigation of communities by reconstruction of unobserved states) using online Galaxy version^4^. The multiple linear regression model that included Brahman proportion of heifers, age in days, and interaction between Brahman proportion and age in days as three independent variables and the relative abundance of microbial genes involved in each metabolic pathways as a dependent variable, was applied. At the same time, the relative abundances of metabolic pathways using pooled samples of BG1 and BG6 were analyzed using the shotgun metagenomics sequencing data to confirm the PICRUSt microbial functional prediction.

**FIGURE 6:**
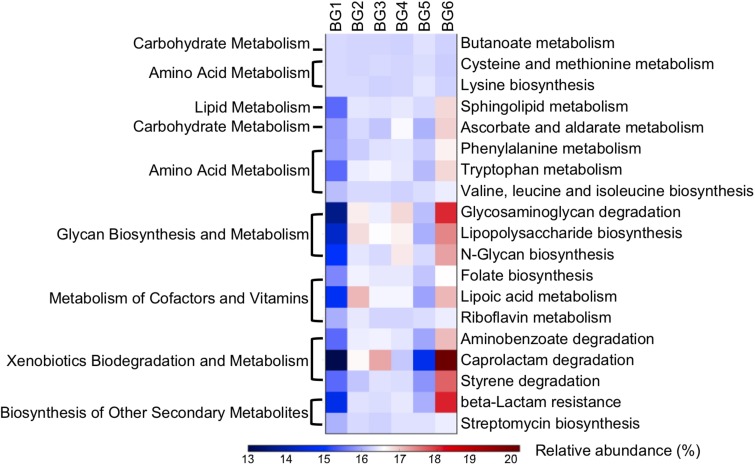
A heatmap showing the difference in relative abundances of the microbial functional genes involved in KEGG pathways among six BGs. Only KEGG pathways with their relative abundance significantly correlated with breed composition (*P* < 0.05) predicted by PICRUSt and consistent with shotgun metagenomics data were involved in the heatmap.

Nineteen metabolic pathways had significant associations with breed composition (*P* < 0.05) and were consistent with differences between BG1 and BG6 pooled sample (Figure 6, Supplementary Tables S6, S7). Among the metabolic pathways for carbohydrates, lipids, and amino acids, bacterial genes involved in amino acid metabolism were the most significantly linearly associated with breed composition, which is likely due to the differences in utilization of proteins between Angus and Brahman. Bacterial genes involved in cysteine and methionine metabolism and lysine biosynthesis were enriched in postweaning heifers with higher Angus proportion, while bacterial genes involved in phenylalanine and tryptophan metabolism, valine, leucine, and isoleucine biosynthesis had higher relative abundance in heifers with greater Brahman proportion. Moreover, microbial genes involved in numerous glycan biosynthesis and metabolism, metabolism of cofactors and vitamins, as well as xenobiotics biodegradation and metabolism pathways showed significant positive linear association with Brahman proportion (Figure 6).

### Effects of Breed Composition on β-Lactam Resistance

Notably, β-lactam resistant genes were predicted to have significantly higher abundance in heifers with more Brahman proportion (Figure 6, *P* < 0.000144). In addition, the relative abundance of genes involved in streptomycin biosynthesis (Figure 6, *P* = 0.00687) also showed a significant positive correlation with Brahman proportion. These results suggest that breed composition influences the bacterial phylogeny that may lead to variation in antimicrobial resistance profiles. To further identify specific β-lactam resistant genes that were enriched in postweaning heifers with more Brahman proportion, associations between the relative abundance of KO functional orthologs, belonging to the β-lactam resistance pathways, and breed composition was analyzed. Among the 13 KO functional orthologs belonging to the β-lactam resistance pathway predicted by PICRUSt among the 95 heifers, the relative abundance of three orthologs showed significant positive linear associations with Brahman proportion. These three orthologs include genes encoding β-lactamase class C (ampC, K01467) (*P* = 0.0002), membrane fusion protein belonging to the multidrug efflux system (arcA, K03585) (*P* = 0.0001), and penicillin-binding protein 2 (K05515) (*P* = 0.0465) (Supplementary Table S5).

**FIGURE 7:**
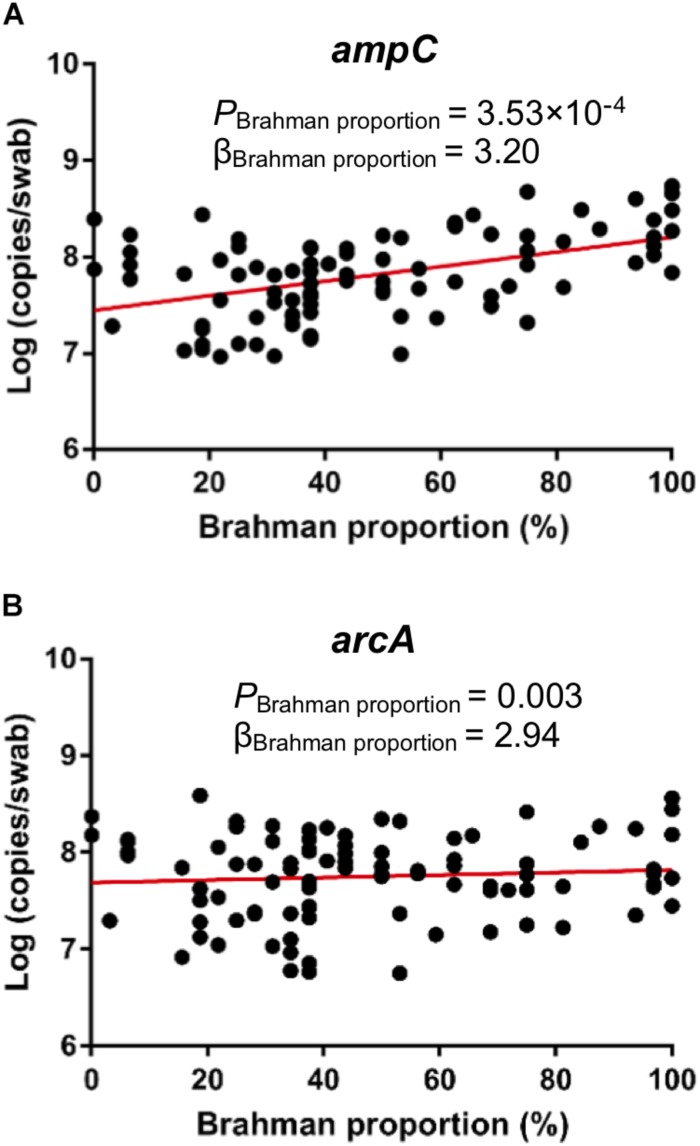
Associations between breed composition and the concentration of ARGs detected by qPCR. Brahman proportion is significantly positively associated with copy numbers of ampC gene **(A)** and arcA gene **(B)** per swab.

qPCR was conducted to detect the concentration of ampC and arcA genes that were predicted to be associated breed composition. Consistent with PICRUSt prediction, copies per swab of both ampC gene (Figure 7A, *P* = 0.0004) and arcA gene (Figure 7B, *P* = 0.0031) showed positive linear associations with Brahman proportion.

**FIGURE 8:**
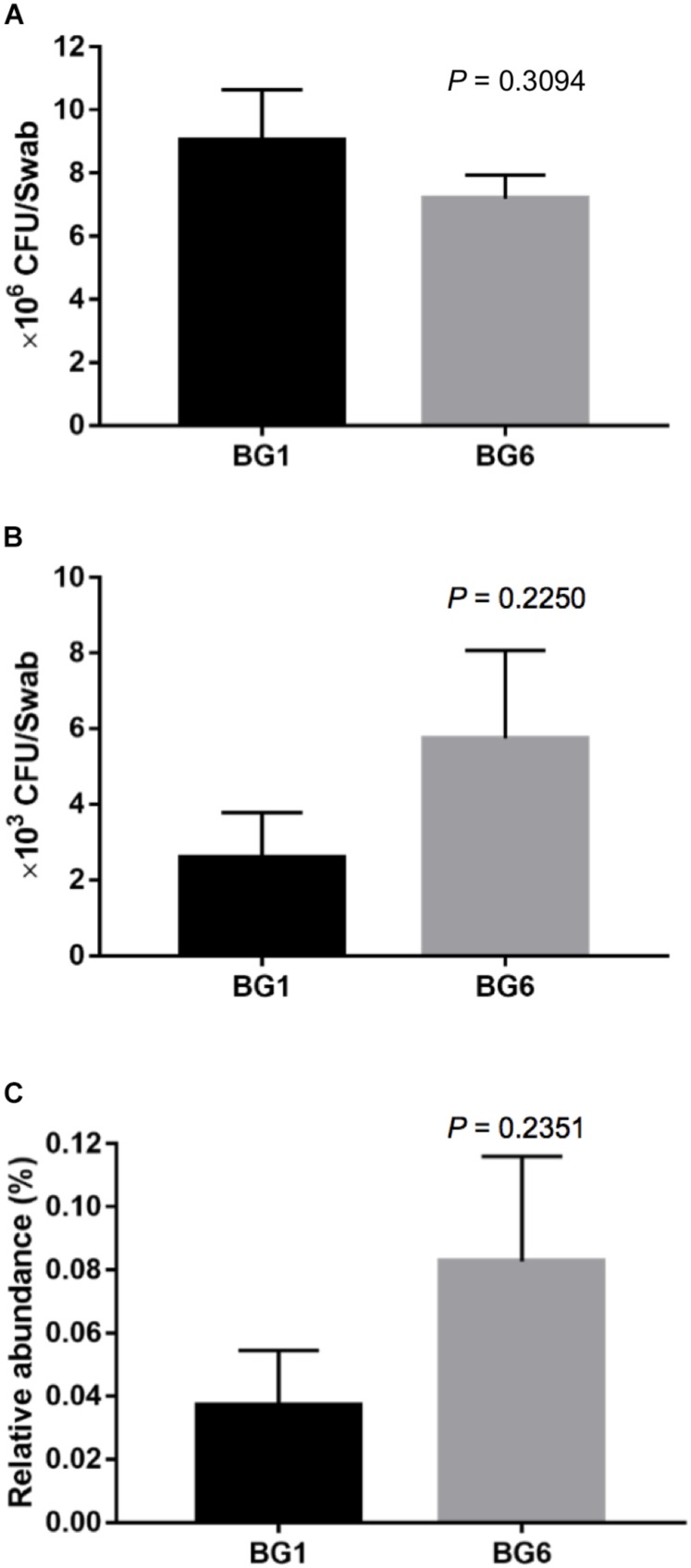
The concentration of ampicillin resistant bacteria (ARB). The concentration of total bacteria **(A)**. The absolute concentration of ARB **(B)**. The relative abundance of ARB **(C)**.

To detect whether β-lactam resistant bacteria were enriched in postweaning heifers with more Brahman proportion, we detected the concentration of culturable ARB in fecal samples from BG1 and BG6 using selective media. The total bacterial concentration was similar between BG1 and BG6 (Figure 8A). The absolute number and the relative abundance of culturable ARB in the fecal samples of BG6 was as twice as of ARB in BG1 (Figures 8B,C, *P* = 0.2250).

## Discussion

Our study investigated the association between breed composition and the hindgut microbiota structure of postweaning heifers and its association with animal growth and possible impact on the antimicrobial resistance profile. We observed that heifers with higher Angus proportion contained more intramuscular fat compared to those with higher Brahman proportion when fed with the same diet, suggesting differences in nutrient digestion and energy harvest among the MAB heifers. Consequently, the hindgut microbiota composition which plays a role in fermentation and digestion was associated with breed composition and animal growth. The variation in hindgut microbiota further results in higher relative abundance of beta-lactam resistance genes in heifers with more Brahman proportion.

Fermentation in the hindgut provides 10 to 15% of the gross energy available to adult cattle (Delaval, 2002). Though we collected fecal samples from the recto-anal junction, the fecal microbiota is similar to colonic and cecal microbiota in terms of phylogenetic distance and bacterial community structure (de Oliveira et al., 2013). Therefore, hindgut microbiota that is associated with breed composition in this study may influence energy harvest and animal growth of the MAB heifers. For example, *Akkermansia*, which was enriched in postweaning heifers in this study (Figure 4F), has been reported to reduce fat-mass by altering the adipose tissue metabolism in a mice model (Everard et al., 2013). Butyrate-producing *Faecalibacterium*, which was positively linearly associated with Angus proportion in this study (Figure 4F), is associated with obesity in human studies (Balamurugan et al., 2010; Karlsson et al., 2013).

Furthermore, we identified several bacterial taxa that have a significant linear association with weight gain and intramuscular fat content (Table 1). For example, Dehalobacteriaceae that were enriched in postweaning heifers with greater weight gain in our study (Table 1) have been reported to be positively correlated with intestinal butyrate level in rats (Casanova-Marti et al., 2018). Butyrate is a major energy source for the colonic epithelium (Hamer et al., 2008). However, further studies are needed to understand whether Dehalobacteriaceae in the GI tract directly improve the growth rate of postweaning cattle through the enhancement of the intestinal butyrate level. The relative abundance of *Succiniclasticum* showed a positive linear association with intramuscular fat content (Table 1) but was not associated with breed composition. Petri et al. (2018) found that *Succiniclasticum* was increased in the rumen when cows were fed with high concentration diet and were positively correlated with fatty acid profiles in subcutaneous fat. Therefore, these bacteria likely contribute to animal growth and fat deposition but are not directly associated with breed composition.

The interaction among host genetics, specific gut bacteria, and host energy expenditure found in this study indicates a potential strategy to improve animal growth and fat content by changing the hindgut microbiota structure. Microbial transplantation has been considered promising to modulate microbiota. However, the Food and Drug Administration (FDA) recently suspends clinical trials involving fecal transplants due to serious antibiotic-resistant infections (FDA, 2019). In addition, many studies reported that bacteria introduced through ruminal or fecal transplantation did not colonize in the GI tract stably, as recipients’ microbiota structure returned to the original status because of host factors (Weimer et al., 2010; Pamer, 2014; Zhou et al., 2018). However, our study suggests that selection for breeds carrying specific bacteria that is associated with desirable phenotype could be promising strategy to shape the hindgut microbiota.

On the other hand, bacteria-bacteria interactions also influence the microbial community structure. Unclassified Lachnospiraceae and unclassified Victivallaceae were the keystone members of the hindgut bacterial co-occurrence network among the 95 MAB heifers. However, the relative abundances of these two bacterial taxa were not associated with breed composition. It has been reported that ruminal unclassified Victivallaceae is associated with genotype of SNP: rs41911152, while ruminal unclassified Lachnospiraceae is associated with genotype of SNP: rs109961459 (Li et al., 2019). Therefore, other host genetic factors beyond breed composition, such as variance in SNP may also shape the gut microbiota mediated by bacterial-bacteria interactions.

The analyses of microbial function indicated that the relative abundances of bacterial genes involved in various metabolic pathways were linearly associated with breed composition. Brahman cattle can maintain high intake levels of low-quality feeds that are deficient in true protein and low insoluble carbohydrates compared to Angus cattle (Hunter and Siebert, 1985; Hegarty, 2004). This can partly explain the higher relative abundance of genes involved in amino acid metabolism, as well as glycan biosynthesis and metabolism pathways observed in the hindgut microbiota of Brahman heifers (Figure 6). Existing variation in digestion and metabolism of proteins and indigestible fibers in hosts with different proportions of Angus and Brahman may yield animals with hindgut environments suitable for bacteria with superior ability for substrate biosynthesis and metabolism.

The MAB heifers in this study were kept on pasture in the same pen after weaning and fed an identical diet with no antibiotics. However, surprisingly the hindgut microbiota in heifers with greater Brahman proportion was predicted to contain more β-lactam resistant genes than heifers with higher Angus proportion (Figures 6, 7). β-Lactam antibiotics are the most widely prescribed group of antibiotics worldwide (Handal and Olsen, 2000), which targets the penicillin-binding proteins (PBPs) and interfere with the structural crosslinking of peptidoglycans to inhibit the biosynthesis of bacterial cell walls (Tenover, 2006; Lin et al., 2015). Here, genes encoding β-lactamase class C, penicillin-binding protein 2, and membrane fusion protein, involved in multidrug efflux system, were predicted to be enriched in heifers with greater Brahman proportion. AmpC β-lactamases are encoded in the chromosomes of many Enterobacteriaceae and a few other organisms, inactivating β-lactam antibiotics by hydrolyzation of the β-lactam rings (Jacoby, 2009). The unclassified Enterobacteriaceae enriched in Brahman heifers may contribute to the higher relative abundance of the *amp*C gene. β-Lactam resistant can also be achieved through the overproduction of PBPs. PBPs are ubiquitous bacterial enzymes that catalyze the polymerization of the glycan strand and cross-linking between glycan chains, which are the final steps of the synthesis of peptidoglycan, the major component of bacterial cell walls (Lovering et al., 2012). PBPs bind to penicillin and other β-lactam antibiotics due to the structural resemblance with pentapeptide precursors that form the peptidoglycan (Sauvage et al., 2008). Interestingly, genes involved in the pathways of glycan biosynthesis and metabolism, particularly lipopolysaccharide and N-glycan biosynthesis were associated with by breed composition (Figure 6), suggesting that the differences in the PBPs gene abundance are likely related to variation in glycan biosynthesis. Further studies are needed to show if the level of antibiotic resistance genes, shown by the shotgun metagenomic sequencing data, are positively correlated with increased expression of enzymes that pose a higher risk of developing β-lactam resistant bacteria. Ampicillin belongs to the β-lactam antibiotics, and is one of the strong inducers and good substrates for ampC β-lactamase (Jacoby, 2009). Interestingly, the postweaning heifers in BG6 that are predicted to contain higher relative abundance of β-lactam resistant genes harbored one fold more culturable ARB compared to those in BG1. As the MAB postweaning heifers were housed together without treatment with antibiotics, these results again suggested that the GI environment of Brahman cattle help enrich the β-lactam resistant bacteria. Further analysis using techniques covering unculturable bacteria, such as functional metagenomics, would be needed to understand the natural GI tract antibiotic resistome.

In summary, understanding of the hindgut microbiota structure of calves with a graduated change of genetic background and desirable traits provides insights for improving animal growth performance by regulation of certain hindgut commensal bacteria. Moreover, host genetics may serve as a driving force shaping the gastrointestinal environment and gut microbiota that may influence on antimicrobial resistance profiles. Therefore, the hindgut microbiota of beef cattle may serve as a new target to improve animal growth, meat quality, and mitigate antimicrobial resistance.

## Data Availability

The 16S rRNA gene sequencing data and the shotgun metagenomics data generated and analyzed in this study are available in the NCBI primary data archive (PDA) with the BioProject PRJNA398505 and PRJNA540379, respectively.

## Ethics Statement

This study followed the protocol for animal care and use approved by the University of Florida Institutional Animal Care and Use Committee (IACUC Number 201408629).

## Author Contributions

PF, ME, CN, and KJ designed the study and wrote the manuscript. PF and JD collected the samples. PF performed the analyses. KJ acquired funding.

## Conflict of Interest Statement

The authors declare that the research was conducted in the absence of any commercial or financial relationships that could be construed as a potential conflict of interest.

## Footnotes

1 https://www.arb-silva.de/documentation/release-132/

2 http://gephi.org

3 http://galaxy.morganlangille.com/

4 http://galaxy.morganlangille.com/

## Supplementary Material

FIGURE S1 The difference in the gut microbiota composition of the postweaning heifers between BG1 and BG6. The PCoA plot showing the separation of the postweaning heifers in BG1 and BG6 based on weighted UniFrac community distance compared by analysis of similarities (ANOSIM).

TABLE S1 Age and breed information of 95 multibreed Angus-Brahman heifers and their dams and sires.

TABLE S2 Guaranteed analysis of UF Custom Range Mineral.

TABLE S3 Information of primers for qPCR.

TABLE S4 The OTU prevalence in six BGs. Only OTUs with the significant prevalence correlated with breed composition were included in the table.

TABLE S5 Influence of heifer’ breed composition, age in days and interactions of these two variables on the relative abundance of core bacterial families and genera. Only core bacterial taxa were included in the table.

TABLE S6 Influence of heifer’ breed composition, age in days and interactions of these two variables on the relative abundance of genes involved in microbial metabolic functional pathways (KEGG 3 level) as well as orthologs involved in beta-lactam resistance pathway (KEGG 4 level) predicted by PICRUSt.

TABLE S7 The counts of genes involved in microbial functional pathways analyzed by shotgun metagenomic sequencing after normalization.
