# Supplementary figures and images for: Animal Breed Composition Is Associated With the Hindgut Microbiota Structure and β-Lactam Resistance in the Multibreed Angus-Brahman Herd
Source: Front Microbiol. 2019 Aug 13;10:1846. doi: 10.3389/fmicb.2019.01846 (PMC6700273; doi:10.3389/fmicb.2019.01846)

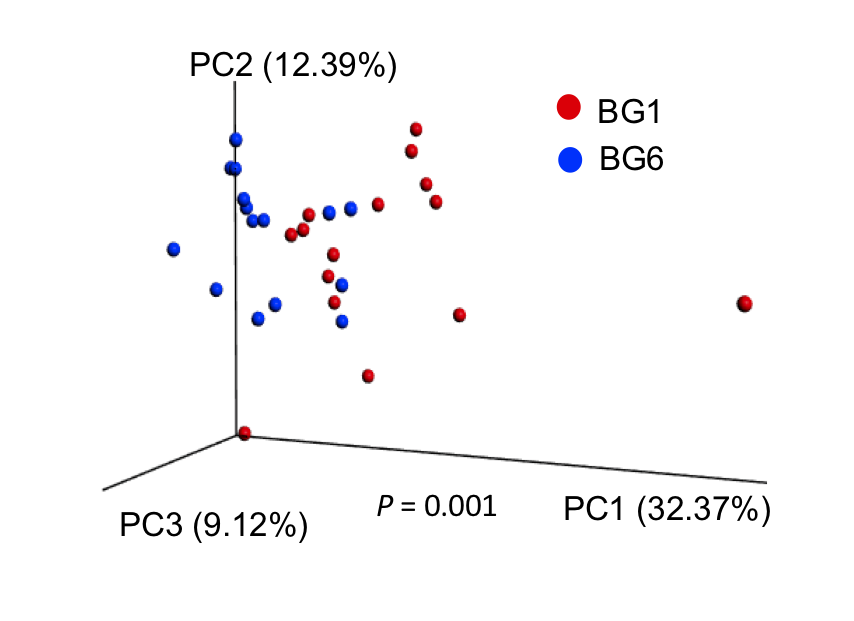

Supplement: FIGURE S1 — The difference in the gut microbiota composition of the postweaning heifers between BG1 and BG6. The PCoA plot showing the separation of the postweaning heifers in BG1 and BG6 based on weighted UniFrac community distance compared by analysis of similarities (ANOSIM). [file Image_1.TIFF]
